# Posttraumatic orientation to bodily signals: The engraving of trauma in bodily perceptions

**DOI:** 10.1192/j.eurpsy.2023.1018

**Published:** 2023-07-19

**Authors:** N. Tsur, Z. Solomon

**Affiliations:** Tel Aviv University, Tel Aviv, Israel

## Abstract

**Introduction:**

Theoretical perspectives emphasize that trauma and complex/posttraumatic stress disorder (C/PTSD) may interrupt with the perception of normal day-to-day bodily sensations, such as hunger, temperature and pain. Yet, a coherent conceptual synthesis of such processes is still lacking.

**Objectives:**

This presentation portrayes two studies that provide empirical grounding for the conceptualization of ‘Posttraumatic Orientation to Bodily Signals’ (posttraumatic-OBS); an umbrella term reflecting the tendency to interpret bodily signals as catastrophic and frightful following trauma.

**Methods:**

Two studies assessing exposure to trauma, C/PTSD, and OBD (Pain catastrophizing scale, PCS; body vigilance scale, BVS; Anxiety sensitivity index-physical), were conducted to test the hypothesized association between exposure to trauma and posttraumatic-OBD, as explained by C/PTSD.

**Results:**

Study 1 included 59 ex-prisoners of war and 44 controls along three time-points, revealing that exposure to trauma was associated with a more catastrophic OBS (t = 2.73, p = .008; Cohen’s d = .57), which was mediated by longitudinal hyperarousal PTSD symptoms (indirect effect = .04 [.009, .11]). Additionally, a long-term chronic trajectory of PTSD was implicated in a more catastrophic OBS (F (2102)=6.91, p = .046).

**Study 2** included 194 dyads of mothers and their young adult daughter. Dyadic path analyses demonstrated that OBD was associated with exposure to trauma, through the mediation of CPTSD among mothers (indirect effects between 0.13–0.28; *p* > 0.021) and daughters (indirect effects between 0.21–0.11; *p* > 0.032). Mothers’ OBD was associated with daughters’ OBD (effects between 0.19-0.27; *p* < 0.016). Daughters’ OBD was serially associated with mothers’ trauma exposure through mothers’ CPTSD and mothers’ OBD, (indirect effect = 0.064; *p* = 0.023). The findings demonstrate that trauma is often implicated in posttraumatic-OBD, which is mediated by C/PTSD, and that these processes may be intergenerationally transmitted.

**Conclusions:**

The findings lay the foundation for the conceptualization of posttraumatic-OBD. The implications of the unified encapsulation of posttraumatic-OBD as an umbrella term reflecting subjective perception of bodily sensations for future research and practice will be presented.

**Disclosure of Interest:**

None Declared

